# Single-Cell Analysis Reveals the Cellular and Molecular Changes of Liver Injury and Fibrosis in Mice During the Progression of *Schistosoma japonicum* Infection

**DOI:** 10.3390/cimb46110707

**Published:** 2024-10-23

**Authors:** Julu Lu, Xinyue Zhang, Panpan Dong, Congjin Mei, Yingying Yang, Chuanxin Yu, Lijun Song

**Affiliations:** National Health Commission Key Laboratory of Parasitic Disease Control and Prevention, Jiangsu Provincial Key Laboratory on Parasite and Vector Control Technology, Jiangsu Provincial Medical Key Laboratory, Jiangsu Institute of Parasitic Diseases, Wuxi 214064, China; 17351645117@163.com (J.L.); zhangxinyue4896@outlook.com (X.Z.); dongpanpan@jipd.com (P.D.); meicongjin@jipd.com (C.M.); yangyingying@jipd.com (Y.Y.)

**Keywords:** single-cell RNA sequencing, *Schistosoma japonicum* infection, liver injury, liver fibrosis, cellular and molecular events

## Abstract

Schistosomiasis is a parasitic disease that poses a serious threat to human health. However, the pathogenic mechanism during the progression of *Schistosoma japonicum* infection remains unclear. In order to elucidate this mechanism, we used single-cell RNA sequencing (scRNA-seq) to investigate the transcriptome characteristics of the cellular (single-cell) landscape in the livers of mice infected with *Schistosoma japonicum*, which were divided into three groups: uninfected mice (0 week (w)), infected mice at 6 w post-infection (the acute phase), and infected mice at 10 w post-infection (the chronic phase). A total of 31,847 liver cells were included and clustered into 21 groups. The cells and T-cells had high heterogeneity in the liver during the progression of schistosome infection. The number and intensity of the intercellular interactions significantly increased at 6 w after infection but decreased at 10 w. The inflammatory signaling pathways chemoattractant cytokine ligand (CCL)5-chemokine C-C-motif receptor (CCR)5 between macrophages and T-cells were predominant at 6 w post-infection; the CCL6-CCR2 signaling pathway between macrophages was predominant at 10 w. The CD80 signaling pathway related to T-cell activation was increased at 6 w after infection, and increased expression of its receptor CD28 on the surfaces of CD4^+^ and CD8^+^ T-cells was confirmed by flow cytometry, suggesting an increase in their activation. In addition, scRNA-seq and quantitative reverse transcription polymerase chain reaction (qRT-PCR) confirmed that the intercellular communication between secretory phosphoprotein 1 (SPP1)-cluster of differentiation (CD44), insulin-like growth factor (IGF)-1-IGF1r and visfatin-insulin receptor (Insr) associated with bone metabolism and insulin metabolism was increased and enhanced in the liver at 6 w post-infection. Overall, we provide the comprehensive single-cell transcriptome landscape of the liver in mice during the progression of schistosome infection and delineate the key cellular and molecular events involved in schistosome infection-induced liver injury and fibrosis. The elevated CCL5-CCR5 and CCL6-CCR2 signaling pathways in the liver may be a drug target for liver injury and fibrosis caused by schistosome infection, respectively.

## 1. Introduction

Schistosomiasis is a parasitic disease that poses a serious threat to human health, which is widespread in tropical and subtropical regions [1]. It is estimated that 200 million people are infected every year around the world, and approximately 200,000 die from schistosomiasis [2]. Adult schistosomes parasitizing the mesenteric veins lay eggs that enter the liver with the portal vein blood flow and remain in the liver tissue, inducing granulomatous inflammatory reactions in the host. Chronic inflammation leads to liver fibrosis, which can further develop into irreversible cirrhosis and liver cancer. However, the pathogenic mechanism during the progression of *Schistosoma japonicum* (*S. japonicum*) infection remains unclear. Therefore, understanding the key cellular and molecular events during the progression of schistosome infection can help control the liver injury and fibrosis caused by schistosome infection [3].

The exocrine proteins secreted by schistosome eggs are engulfed by macrophages, which are processed and presented to helper T-cells (Th cells) to activate these cells, which then secrete more cytokines; macrophages, eosinophils, neutrophils, and plasma cells are attracted to and concentrated around the eggs to form granulomas around the eggs [4]. As the tissue damage is repaired, hepatic stellate cells are activated and release extracellular matrix, leading to the development of liver fibrosis [5]. In the early stages of schistosome infection, the Th1 immune response predominates, resulting in the secretion of the tumor necrosis factor (TNF)-α, interleukin (IL)-1, interferon (IFN)-γ and IL-2 cytokines and the promotion of inflammatory responses [6]. This response gradually switches to a Th2 response 4 weeks (w) after infection, with the secretion of IL-4, IL-5, and IL-13, which inhibit overactivation of the inflammatory reaction and stimulate the formation of egg granulomas and fibrosis. An imbalance in the Th1/Th2 cell ratio is closely related to the progression of hepatic fibrosis [7]. M1-type macrophages (CAMs), which secrete iNOS, promote the Th1-type response and inhibit fibrosis; M2-type macrophages (AAMs), which secrete IL-10 and Arg1, promote the Th2-type response and promote fibrosis [8]. In addition, various cells in the liver, including natural killer cells, B cells, and hepatic stellate cells, play a key role in schistosomiasis [3,9,10].

To elucidate the roles and trends of immune cells and key molecules expressed during the progression of schistosome infection, we used single-cell RNA sequencing (scRNA-seq) to investigate the transcriptome characteristics of the cellular (single-cell) landscape in the livers of mice infected with *Schistosoma japonicum* at different stages: uninfected (0 week (w)), the acute phase (6 w post-infection), and the chronic phase (10 w post-infection). The results, combined with quantitative reverse transcription polymerase chain reaction (qRT-PCR) and flow cytometry analysis, showed that the CCL5-CCR5 and CCL6-CCR2 signaling pathway was elevated in the acute phase and chronic phase, respectively. In addition to the various subsets of CD4^+^ T-cells, CD8^+^ T-cells may also be involved in the liver injury and fibrosis of schistosomiasis. These results are helpful for elucidating the formation and outcome of liver pathological damage caused by schistosomiasis.

## 2. Materials and Methods

### 2.1. Animals

Female C57BL/6 mice (6–8 w), weighing 15–25 g (SPF), were obtained from Sibeifu Biotechnology Co., Ltd., Suzhou, China and housed at the Jiangsu Institute of Parasites Diseases in compliance with international and national standards. The use of mice was approved by the Ethics Review Committee of the Jiangsu Institute of Parasites Diseases, with approval ID number JIPD-2023-001. Infected snails were obtained from the Snail Department of Jiangsu Institute of Parasites Diseases.

### 2.2. Constructing a Mouse Model of Infection with S. japonicum

Each mouse was infected with 15 ± 1 cercariae, and the mice were sacrificed at 6 and 10 w after infection. The mouse livers were collected for pathological section staining and scRNA-seq, and healthy uninfected mice were used as controls.

### 2.3. Histopathology

The liver tissue was fixed, dehydrated and embedded in a routine manner. A thickness of 4 µm was used for conventional dewaxing to water, and the samples were stained via HE and Masson’s detection kits from Solaibio Science & Technology Co., Ltd (Beijing, China). On average, 8 ± 2 slices, selected from each group, were photographed using cellSens under an upright microscope, and the area sizes of the single egg granuloma and collagen fiber were calculated.

### 2.4. scRNA-Seq

Liver tissue samples from one healthy uninfected mouse and one mouse infected with *S. japonicum* at 6 w and 10 w were collected and washed in precooled PBS. The liver was digested with 1 mg/mL collagenase, and a single-cell suspension was prepared by passing the sample through a 40 μm sieve. The construction and sequencing of the single-cell library were conducted by OE Biotech Co., Ltd. (Shanghai, China). The liver tissue was freshly collected and divided into two parts, one for single-cell dissociation and the other temporarily stored in tissue protective solution (MACS, 130-100-008, Cologne, Germany) at 4 °C for nuclear preparation. Single-cell dissociation was first performed to ensure that the single-cell suspension preparation was finished at the same time as the nucleus suspension preparation. The tissues used for single-cell dissociation were washed twice with precooled medium (DMEM + 0.04% BSA) and fully minced into pieces, followed by digestion with enzyme solution (DMEM + 0.04% BSA + 0.2% collagenase II (Gibco, Grand Island, NY, USA) + 100 μg/L DNase I (AppliChem, Gatersleben, Germany)) at 37 °C and 22 rpm for 20–30 min and then 0.25% trypsin (Gibco, Grand Island, NY, USA) for 5 min. The cell suspensions were filtered with a 40 μm cell sieve and then subsequently subjected to centrifugation at 4 °C and 300× *g* for 7 min. Erythrocytes were lysed via MACS Red Cell Lysis Solution (130-094-183). After washing with medium, the cell precipitate was resuspended in 100–200 μL of PBS + 1% BSA. When single cells were centrifuged after erythrocyte lysis, the preparation of the nuclei with another tissue was started. The tissues were washed twice with precooled medium, sheared into millet, and then lysed with 1 mL of precooled NST for 3–5 min. The nuclear suspension was filtered through a 40 µm cell sieve, then centrifuged at 4 °C for 10 min at 500× *g*. The precipitate was washed with 2 mL of wash buffer and centrifuged at 4 °C for 5 min at 500× *g*. The cell nuclei were resuspended in 100 µL of PBS + 1% BSA + 1 U/μL RNase inhibitor. The nuclei were stained with Trypan blue and counted via microscopy. The concentration was adjusted to 700–1200 cells/μL, and the mixture was kept on ice before use.

A 10× Genomics Chromium Next GEM Single Cell 3′ Kit v3.1 (1000268) was used for the scRNA-seq according to the manual (CG000315). The single cells and the nuclei were mixed equally and immediately loaded on a chip to run on a 10× Chromium Controller for GEM formation. Reverse transcription, cDNA amplification and DNA library construction were carried out in accordance with the protocol. The concentration and fragment size of the libraries were determined separately with an Invitrogen Qubit 4.0 (Thermo Fisher Scientific, Massachusetts, USA) and an Agilent 4150 TapeStation (Agilent Technologies, California, USA). PE-150 mode was used for the high-throughput sequencing.

### 2.5. Analysis of Single-Cell RNA-Seq Data

The FASTQ files were analyzed using MobiVision software (version 2.1), and the unique molecular identifier (UMI) counts were summarized for the different mRNA in each cell, aligned to the mm10 mouse reference genome, and subjected to further analysis. The UMI counts were subsequently evaluated using Seurat R (version 4.0.0) [11]. Cells with a gene count >200, a UMI count >1000, a log10GenesPerUMI >0.7, a mitochondrial UMI percentage <5%, and a hemoglobin gene percentage <5% were retained as high-quality cells. The DoubletFinder [12] package (version 2.0.3) was used to remove doublet cells. Once the quality control was completed, the data were normalized using the NormalizeData function in the Seurat package (version 3.1.2).

Using the Find-VariableGenes function (mean.function = FastExpMean, dispersion.function = FastLogVMR), the top 2000 highly variable genes (HVGs) were screened. Principal component analysis (PCA) was used to reduce the dimensionality of the expression profiles of genes with high variability, and the results were visualized in two dimensions by Uniform Manifold Approximation and Projection (UMAP). Marker gene identification was performed using the FindAllMarkers function (test.use = presto) from the Seurat package. The identified marker genes were subjected to visualization via the VlnPlot and FeaturePlot functions. Differential gene screening was performed using the FindMarkers function in the Seurat package (test.use = presto) to screen for differentially expressed genes (DEGs) based on a *p* value < 0.05 and |log2fold change| > 0.58, and Gene Ontology (GO) and Kyoto Encyclopedia of Genes and Genomes (KEGG) enrichment analyses of differentially significant genes were performed by hypergeometric distribution tests using R (version 4.0.3).

### 2.6. Pseudotime Analysis and Cell Communication Analysis

The data in Seurat were extracted using the importCDS function of the Monocle2 package (version 2.9.0) [13] and a CellDataSet was created. The differentialGeneTest function was used to filter out the differentially expressed genes (sorted genes, qval < 0.01). Reduced dimensional clustering was performed using the reduceDimension function, and the differentiation trajectories were inferred using the orderCells function. Component 1 and component 2 on the horizontal and vertical axes were downscaled components reflecting the position of the cell in the proposed temporal trajectory. The pseudotime reflected the relative order of the cell in development or state transitions. Larger values indicated that the cell was at a more advanced stage of development or differentiation.

To analyze the cell communication information from the single-cell transcriptomics (scRNAseq) data, the CellPhoneDB (version 4.1.0) was used [14]. Genes that were expressed in at least 10% of the cells in a given cell type were selected for analysis, and then the cell type markers of all the cells were randomized to form new cell clusters (randomized 1000 times by default), and the average of the mean expression levels of the ligand in the randomized cell clusters and of the receptor in the cell types with which it interacts were calculated. For each receptor–ligand pair in each pairwise comparison between the two cell types, a null distribution was generated. By calculating the proportion of means equal to or higher than the actual mean, a *p* value was obtained indicating the likelihood of cell type specificity for a given receptor–ligand complex. The cellular communication network was mapped using the R packages Igraph (version 1.2.4.1) and Circlize (version 0.4.8).

### 2.7. qRT-PCR

Before infection (0 w) and 6 and 10 w post infection, the total RNA was extracted from the liver tissues (50 to 100 mg) of five mice with an RNA extraction kit (SparkJade, Jinan, China), respectively. cDNA was subsequently synthesized using a first-strand cDNA synthesis SuperMix kit (TransGen Biotech, Beijing, China). Subsequently, a quantitative PCR was conducted by employing a SYBR Green kit (TransGen Biotech, Beijing, China) for fluorescent analysis. The PCR cycling protocol was as follows: pre-denaturation at 94 °C for 30 s, 45 cycles of 94 °C for 5 s, and 60 °C for 30 s. The 2^−ΔΔCt^ method with GAPDH as the control was used to calculate the relative expression levels. The primer sequences were obtained from GenScript Biotech (Nanjing, China), as indicated in Supplementary Table S1.

### 2.8. Flow Cytometry

Before infection (0 w) and 6 w post-infection, the liver tissues from five mice were digested with 0.02% collagenase and 0.002% DNase I at 37 °C for 30 min, respectively. The digested mixture was subjected to filtration through 70 μm sieves and the single-cell suspensions were prepared. Subsequent to centrifugation at 30× *g* for 5 min, the cellular supernatants were centrifuged in a new tube at 400× *g* for 5 min. The precipitate was resuspended with 40% Percoll solution (3 mL). Next, 70% Percoll (3 mL) was added to another centrifuge tube, and the 40% Percoll cell suspension was carefully overlaid on top of the 70% Percoll solution, which was subjected to centrifugation at 800× *g* for 20 min. After centrifugation, lymphocytes at the interface of the 2-layer Percoll solution were extracted. The lymphocytes (2 × 10^6^) were incubated with the following labeled antibodies: allophycocyanin (APC) anti-CD8, fluorescein isothiocyanate (FITC) anti-CD4, PE/Cyanine7 anti-mouse CD28, Brilliant Violet 421™ anti-mouse CD274 (B7-H1, PD-L1). All the antibodies were from Becton, Dickinson and Company (NJ, USA). Subsequently, the cells in the samples were analyzed by flow cytometry.

### 2.9. Statistical Methods

Data were displayed as the means ± standard deviations. Comparisons between two groups were performed using *t* tests. The statistical analysis was conducted with GraphPad Prism 9.0 software. A *p* value < 0.05 was considered statistically significant.

## 3. Results

### 3.1. Atlas of Liver Cells in Mice During the Progression of S. japonicum Infection

**Figure 1:**
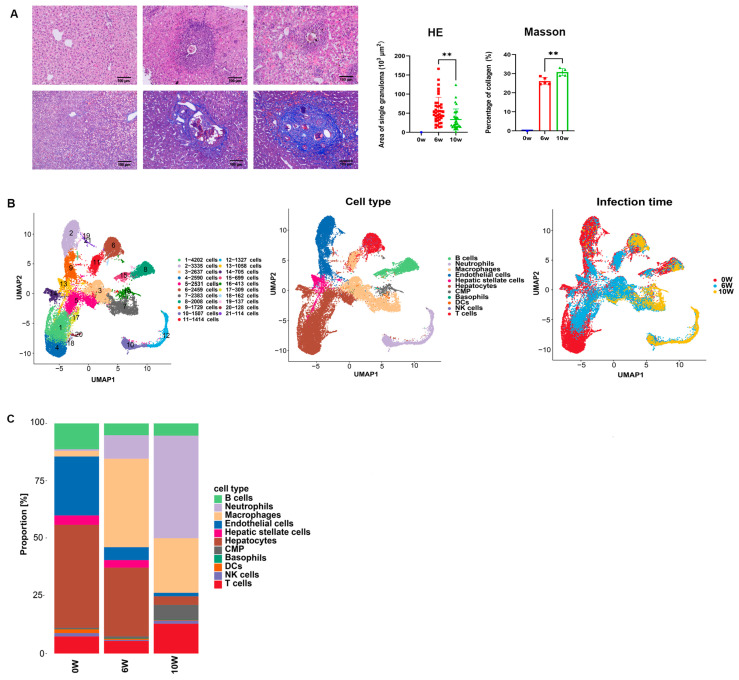
Pathological changes and cell clustering annotation in the livers of mice during the progression of *S. japonicum* infection. (**A**) Liver pathological changes at 0 w (healthy mice), 6 w and 10 w after schistosome infection. (**B**) UMAP graph of the dimensionality reduction clustering of single cells (colored by cluster, cell type, and infection time). (**C**) Changes in the proportion of liver cell populations in different groups before and after schistosome infection. ** *p* < 0.01.

The mice were infected with *S. japonicum* cercariae, and the livers of normal mice before infection (0 w), as well as those of mice 6 and 10 w after infection, were collected for HE and Masson’s pathological section staining. The HE staining results revealed that *S. japonicum* eggs were deposited in the livers of the mice at 6 and 10 w after infection. Egg granulomas were formed around the eggs by immune cells such as macrophages and eosinophils, T cells as well as hepatocytes. At 6 w, the area of the single egg granuloma was significantly larger than that at 10 w. The collagen area (dyed blue) was observed by Masson’s trichrome staining, and the results revealed a large amount of collagen deposition around the eggs. The collagen area in the liver was greater at 10 w than at 6 w. Pathology confirmed the successful establishment of the schistosome infection model in the mice (Figure 1A). The distribution of high-quality cells for quantitative quality control in each sample ranged from 5545 to 20,444, according to the single-cell transcriptome analysis. After the exclusion of two-cell, multi-cell and apoptotic cells, the final distribution of cell numbers ranged from 3634 to 17,638. The number of liver cells in the mice before infection was 17,638 at 0 w, 3634 at 6 w after infection, and 10,575 at 10 w after infection, as obtained in the sample. Following dimensionality reduction clustering, a total of 21 clusters of cells were established (Figure 1B). Eleven cell groups, namely, B cells (8, 15, 19-1 cell clusters, cluster of differentiation (CD)19^+^CD79A^+^CD19B^+^), neutrophils (10, 12 cell clusters, LY6G^+^NCF1^+^Rethlg^+^), macrophages (3, 7 cell clusters, adgre1^+^csf1r^+^MAFB^+^), endothelial cells (2, 9, 19-2 cell clusters, CDH5^+^TM4SF1^+^CLEC14A^+^), hepatic stellate cells (13 cell cluster, Col3a1^+^DCN^+^reln^+^), hepatocytes (1, 4, 5, 14, 17, 18-2, 20 cell clusters, Apob^+^PCK1^+^CYP2E1^+^), myeloid progenitor cells (16 cell cluster, CMP, MPO^+^ELANE^+^prtn3^+^), basophils (19-2 cell cluster, Cyp11a1^+^Ms4a2^+^), dendritic cells (7 cell clusters, Flt3^+^CD1C^+^CST3^+^), NK cells (6, 11 cell clusters, Nkg7^+^Klrd1^+^Klrc1^+^), and T-cells (6, 11 cell clusters, CD3G^+^CD3D^+^CD3E^+^), were established according to the expression of classic cell marker genes (Figure 1B, Supplementary Figure S1). The livers of healthy, uninfected mice are mainly composed of hepatocytes, endothelial cells, and B cells. After 6 w of infection, the livers of the mice were composed mainly of hepatocytes, macrophages, and neutrophils. After 10 w of infection, the livers of the mice were mainly composed of neutrophils, macrophages, and T-cells. The cell types in the liver changed substantially as the disease progressed. The number of macrophages increased significantly at both 6 and 10 w after infection, but the increase was more pronounced at 6 w. While the number of neutrophils gradually increased, the number of hepatocytes, B cells and endothelial cells decreased after infection (Figure 1C).

### 3.2. Changes in T-Cell Subtypes in the Livers of Mice During the Progression of S. japonicum Infection

**Figure 2:**
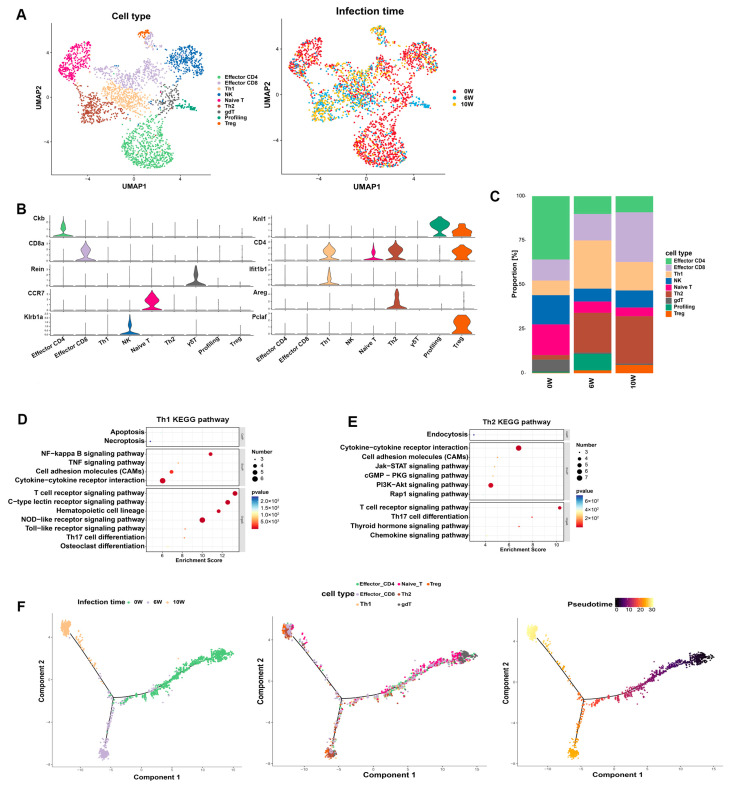
Changes in the T-cell subtypes in the livers of mice during the progression of *S. japonicum infection.* (**A**) The 2322 T-cell dimensionality reduction clustering UMAP plot (stained by cell type and infection time). (**B**) Violin plot of the gene expression in different T-cell subtypes. (**C**) Changes in the T-cell subtypes in the livers of mice before and after schistosome infection. (**D**) Bubble plot of the enriched KEGG pathways in Th1 cells compared with other T-cell subtypes. (**E**) Bubble plot of the enriched KEGG pathways in Th2 cells compared with other T-cell subtypes. (**F**) Pseudotemporal analysis of the T-cell subtypes.

We clustered and classified 2322 T-cells into nine subgroups on the basis of cell-specific marker genes (effector CD4, effector CD8, Th1, NK, naïve T, Th2, γδ T, profiling, and regulatory (Treg) T-cells) (Figure 2A) and the top 10 signature marker genes of each T-cell subpopulation (Supplementary Figure S2). The cell-specific gene expression levels for each subpopulation were determined via violin plots. *Zbtb16* and *Ckb* were used to label the effector CD4 T -cells; *CD8b1* and *CD8a* were used to label the effector CD8 T-cells; *CD4*, *Casp1*, and *lfit1bl1* were used to label the Th1 cells; *Klrb1a* and *Ncr1* were used to label the NK cells; *Ccr7* and *S1pr1* were used to label the naïve T-cells; *Areg* and *ll1rl1* were used to label the Th2 cells; *Reln* and *Bhmt* were used to label the γδ T-cells; *Knl* was used to label the profiling T-cells; and *Pclaf* and *Bub1* were used to label the Treg cells (Figure 2B). The T-cells in the liver before infection were primarily effector CD4^+^ T-cells, effector CD8^+^ T-cells, NK cells, naïve T-cells, and γδ T-cells. The T-cells in the liver at 6 w post-infection were primarily Th1, Th2, effector CD8, and profiling T-cells; the T-cells in the liver at 10 w post-infection were primarily effector CD8, Th1, Th2 and Treg cells. Although the T-cell types at 6 and 10 w post-infection were roughly similar, the proportions were altered, with the proportion of Th1 cells decreasing with the progression of infection, the proportion of Th2 cells increasing, and the proportion of Treg cells increasing in the later stages of infection. Compared with those of the T-cell subtypes in the liver before infection, the proportions of effector CD4 T, NK, naïve T, and γδ T-cells decreased, whereas the proportions of effector CD8 T, Th1, Th2, and Treg cells increased (Figure 2C).

The Th1-enriched signaling pathways included the NOD-like receptor signaling pathway, T-cell receptor signaling pathway, C-type lectin receptor signaling pathway, cytokine–cytokine receptor interaction pathway, and NF kappa B signaling pathway (Figure 2D). The signaling pathways enriched with Th2 cells included cytokine–cytokine receptor interaction, the T-cell receptor signaling pathway, the PI3K/Akt signaling pathway, and Th17 cell differentiation (Figure 2E).

According to the pseudotemporal analysis, the developmental trajectory of cell differentiation revealed that the T-cells at 10 w were located in the opposite direction to those at 0 w, and those at 6 w were located between the two, indicating that the T-cells at 6 w were in an intermediate functional state (Figure 2F). γδ T-cells are enriched in the early stages of pseudotime, confirming their protective role in liver injury [15]. Naïve T-cells gradually differentiate into effector CD4 T- and effector CD8 T-cells. Effector CD4 T-cells gradually differentiate in the Th1, Th2, and Treg directions. The effector CD8 T-, Th1, and Th2 cells were enriched in the middle and late stages of the pseudotemporal sequence, indicating their association with the formation of liver granulomas and fibrosis. The Treg cells were enriched in the late stage of the pseudotemporal sequence, indicating their association with the development of liver fibrosis.

### 3.3. Analysis of Communication Between the Liver Cells in Mice During the Progression of S. japonicum Infection

**Figure 3:**
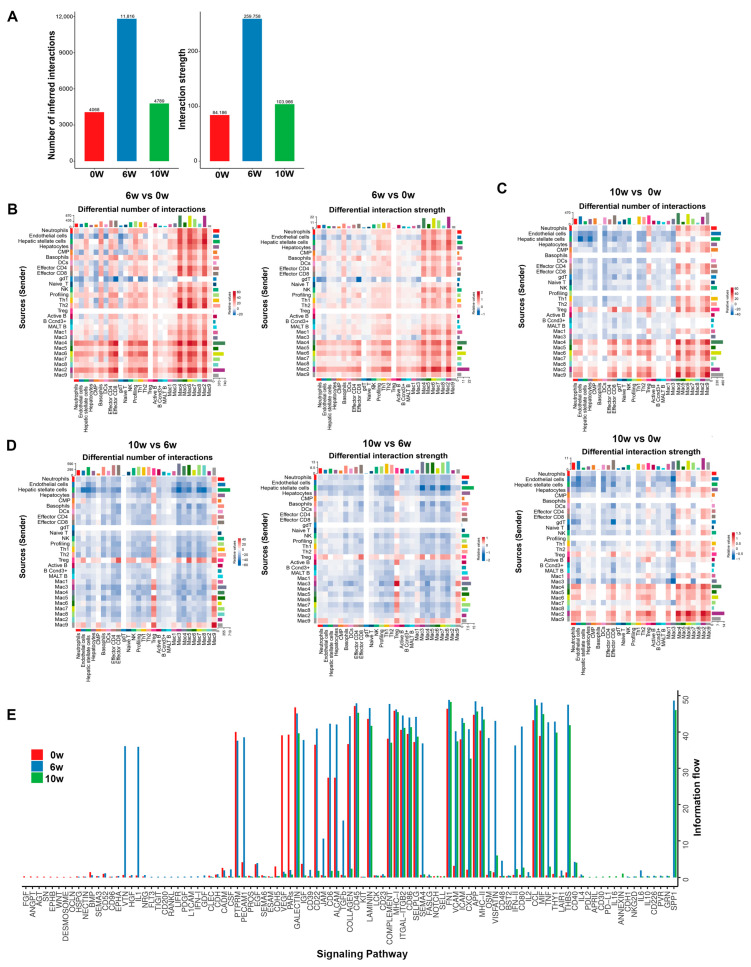
Cell communication in the livers of mice during the progression of *S. japonicum* infection. (**A**) The number and intensity of the interactions between cells in the liver before and after *S. japonicum* infection. (**B**) The number and intensity of the interactions between liver cells in mice infected with schistosomes at 6 w post-infection compared with those in uninfected mice (0 w). (**C**) The number and intensity of the interactions between liver cells in mice infected with schistosomes at 10 w post-infection compared with those in uninfected mice (0 w). (**D**) The number and intensity of the interactions between liver cells in mice infected with schistosomes at 6 w post-infection compared with those in mice at 10 w post-infection. (**E**) Information flow of various signaling pathways in the livers of mice before and after schistosome infection.

To further analyze the interactions between cells, we conducted an intercellular communication analysis and found that the number and intensity of the intercellular interactions were significantly enhanced at 6 w after infection but decreased at 10 w (Figure 3A). Through heatmap analysis, we found that, compared with those of the livers of uninfected mice, the number and intensity of the interactions between the liver cells of infected mice at 6 w and 10 w were increased, with a considerable rise in the number and intensity of the interactions between macrophages and other cells. Among them, clusters 4, 6, 7, and 2 of the macrophages at 6 w had the most interactions, whereas clusters 4, 6, and 9 of the macrophages at 10 w had the most interactions (Figure 3B,C). Compared with that at 6 w after infection, the interaction between Treg cells and other cells in the livers of the infected mice at 10 w was greater, indicating that Treg cells play a major role in disease prognosis in the later stage of schistosome infection (Figure 3D). A comparison of the overall information flow of various signaling pathways revealed that the intercellular signaling pathways in the liver after schistosome infection were enriched in inflammatory signaling pathways, such as the chemoattractant cytokine ligand (CCL), TNF, C-X-C motif chemokine ligand (CXCL), complex, and activated leukocyte cell adhesion molecule (ALCAM) pathways; T-cell activation-related pathways, such as the CD40, CD86, MHCI, II, and THY1 pathways; and fibrosis-related pathways, such as the collagen, laminin, and TGF-β pathways. In addition, the insulin-like growth factor-1 (IGF-1), visfatin, and secretory phosphoprotein 1 (SPP1) signaling pathways were increased after infection with schistosomes. However, the vascular endothelial growth factor (VEGF), proteinase-activated receptors (PARs)-related pathways were suppressed after infection (Figure 3E).

### 3.4. Increased CCL and CXCL Signaling Pathway Activity in the Livers of Mice During the Progression of S. japonicum Infection

**Figure 4:**
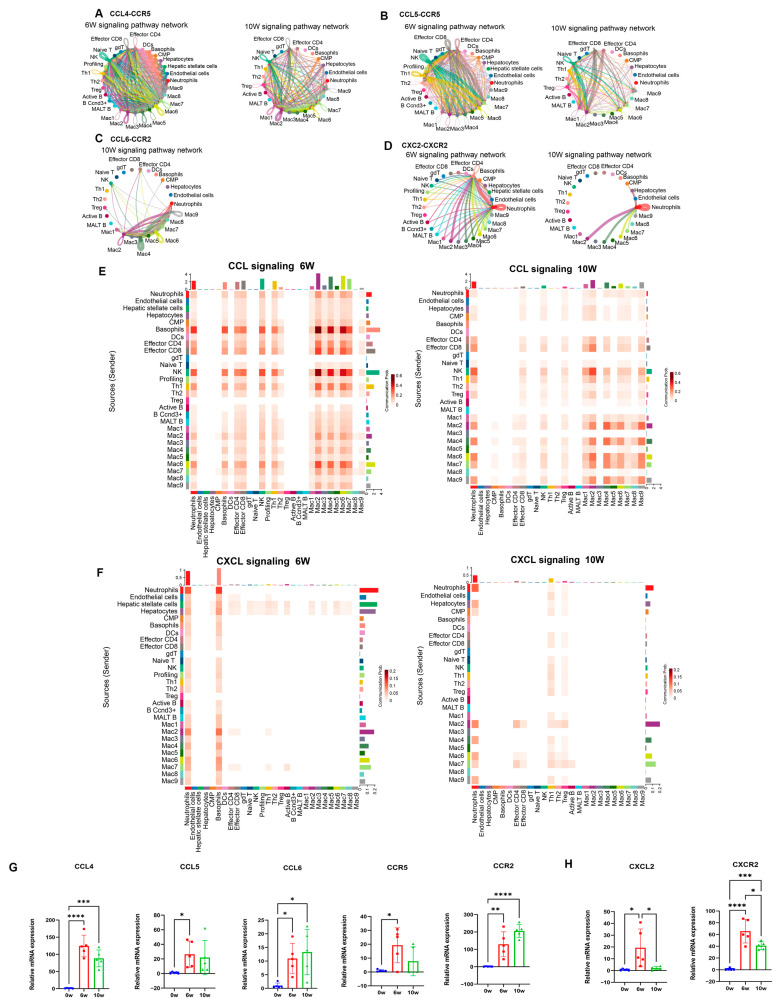
Promoted CCL and CXCL signaling pathways in the livers of mice during the progression of *S. japonicum* infection. (**A**) Cellular communication network diagram of CCL4-CCR5 in the liver at 6 and 10 w after schistosome infection. (**B**) Cellular communication network diagram of CCL5-CCR5 in the liver at 6 and 10 w after schistosome infection. (**C**) Cellular communication network diagram of CCL6-CCR2 in the liver of mice at 10 w after schistosome infection. (**D**) Cellular communication network diagram of CXCL2-CXCR2 in the liver of mice at 6 and 10 w after schistosome infection. (**E**) Heatmap of the intensity of the CCL signaling pathway between different cell types in the liver at 6 and 10 w after schistosome infection. (**F**) Heatmap of the intensity of the CXCL signaling pathway between different cell types in the liver at 6 and 10 w after schistosome infection. (**G**) The relative mRNA expression of CCL4-CCR5, CCL5-CCR5, and CCL6-CCR2 in the liver of mice. (**H**) The relative mRNA expression of CXCL2-CXCR2 in the liver of mice at 0 w, 6 w, 10 w after schistosome infection (n = 5). * *p* < 0.05, ** *p* < 0.01, *** *p* < 0.001, **** *p* < 0.0001.

The distribution of the ligands and receptors revealed that the CCL4-CCR5 and CCL5-CCR5 signaling pathways were mainly present in the livers of the mice at 6 w after infection; the CCL4-CCR5 and CCL6-CCR2 signaling pathways were predominant after 10 w of infection (Supplementary Figure S3A). Network diagrams and cellular communication heatmaps of the related genes revealed that the chemokine CCL increased and was increased between macrophages, neutrophils, NK cells, eosinophils, and Th1 cells, as well as between effector CD4^+^ and CD8^+^ T-cells. Among them, the expression levels of the CCL5, 3, and 4 genes increased in almost all the cells. The expression levels of the CCR5 and 2 receptors mainly increased in macrophages (Figure 4A–C,E), which was confirmed by violin plots (Supplementary Figure S3B). qRT-PCR was used to determine the mRNA expression of CCL in the liver, confirming that the mRNA expression of CCL4-CCR5 and CCL5-CCR5 increased in the livers of the mice at 6 w after infection; CCL6-CCR2 gradually increased after infection and reached its highest expression at 10 w (Figure 4G).

The CXCL signaling pathway was enriched with CXCL2-CXCL2R in the liver at 6 w and 10 w after infection (Supplementary Figure S4A). Through network diagrams, cellular communication heatmaps and violin diagrams of related genes, we found that the expression of genes related to the CXCL signaling pathway increased and also increased between neutrophils, basophils, macrophages, hepatic stellate cells, and hepatocytes. CXCL2 was expressed in almost all the cells, and the receptor CXCL2R was mainly expressed in neutrophils and basophils (Figure 4D,F, Supplementary Figure S4B). qRT-PCR confirmed that the CXCL2-CXCL2R expression increased after infection, with the expression levels at 6 w being greater than those at 10 w (Figure 4H).

### 3.5. CD80 Signaling Pathway Related to T-Cell Activation Signaling Pathways in the Livers of Mice During the Progression of S. japonicum Infection

**Figure 5:**
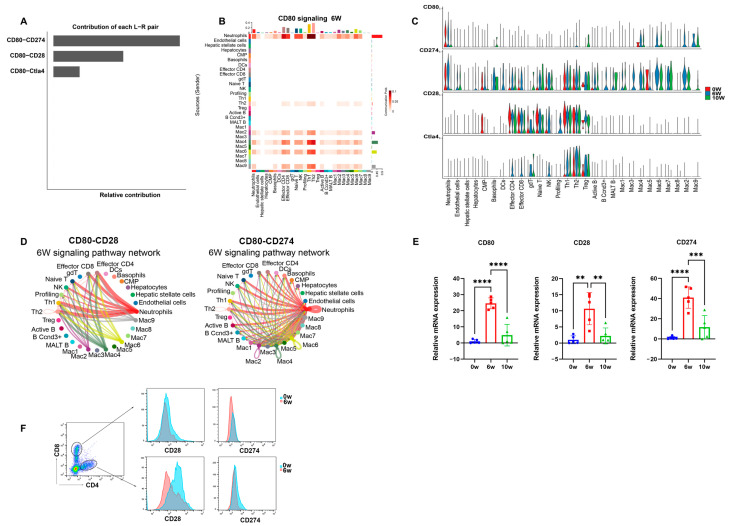
CD80 signaling pathway related to T-cell activation signaling pathways in the livers of mice during the progression of *S*. *japonicum* infection. (**A**) Distribution of CD80 ligands and receptors in the livers of mice at 6 w after schistosome infection. (**B**) Heatmap of the communication intensity of the CD80 signaling pathway between different cell types in the livers of mice at 6 w after schistosome infection. (**C**) Violin plot of the expression distribution of CD80 signaling pathway-related genes. (**D**) Cellular communication network diagram of CD80-CD28 and CD80-CD274 in the livers of mice at 6 w after schistosome infection. (**E**) The relative mRNA expression of CD80-CD28 and CD80-CD274 in the livers of the mice at 0 w, 6 w, 10 w after schistosome infection (*n* = 5). (**F**) Flow cytometry detection of the expression of CD28 and CD274 on CD4^+^ and CD8^+^ cells in the liver of the mice at 0 w, 6 w, 10 w after schistosome infection (*n* = 5). ** *p* < 0.01, *** *p* < 0.001, **** *p* < 0.0001.

According to the cellular communication heatmap and network diagram, the CD80 signaling pathway related to T-cell activation was increased at 6 w after infection. The distribution of the ligands and receptors revealed that the CD80 signaling pathway was mainly composed of CD80-CD274 and CD80-CD28 (Figure 5A). Violin and network diagrams revealed that CD80 molecules related to T-cell activation were mainly expressed in neutrophils, with its receptor CD274 expressed in almost all the cells, whereas CD28 was mainly expressed in Th1 cells and in Th2, effector CD4, and CD8 cells (Figure 5B–D). The mRNA expression levels of CD80, CD274, and CD28 in the liver were increased at 6 w after infection, which was consistent with the sequencing results (Figure 5E). Flow cytometry analysis revealed increases in the expression of CD28 and CD274 in CD4^+^ and CD8^+^ cells in the liver (Figure 5F).

### 3.6. Increased SPP1, IGF-1 and Nampt Signaling Pathway Activity in the Livers of Mice Infected with S. japonicum

**Figure 6:**
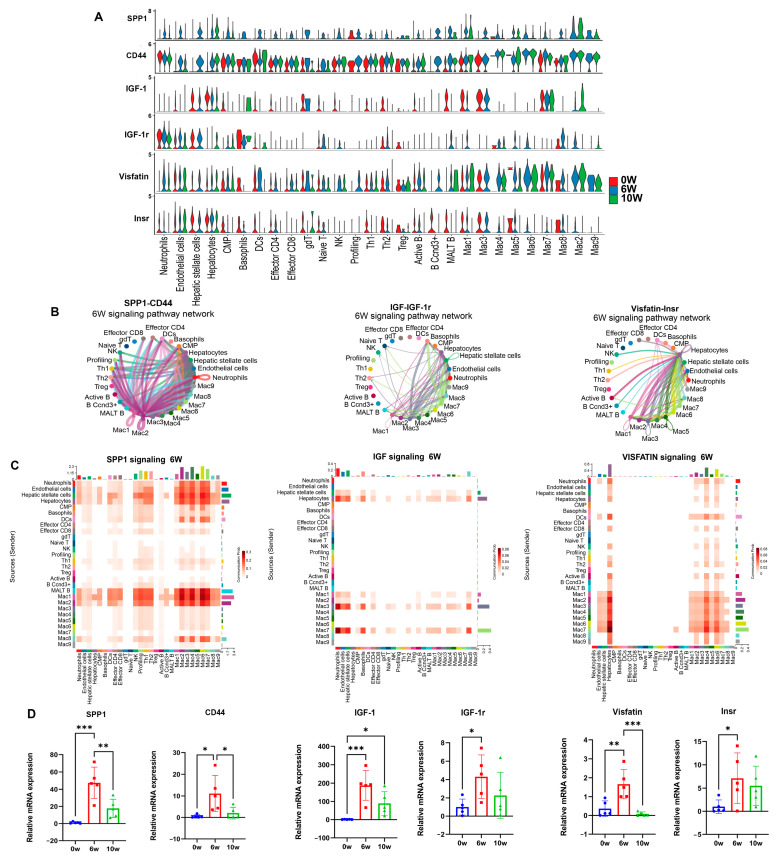
Promoted SPP1, IGF1 and visfatin signaling pathways in the livers of mice infected with schistosomes. (**A**) Violin plot of the expression distribution of SPP1, IGF1 and visfatin signaling pathway-related genes. (**B**) Cellular communication network diagram of SPP1-CD44, IGF-1-IGF-1r and visfatin-Insr in the liver of mice at 6 w after schistosome infection. (**C**) Heatmap of the communication intensity of the SPP1, IGF1 and visfatin signaling pathway between different cell types in the liver of mice at 6 w after schistosome infection. (**D**) The relative mRNA expression of SPP1-CD44, IGF-1-IGF-1r and visfatin-Insr in the liver of mice at 0 w, 6 w, 10 w after schistosome infection (n = 5). * *p* < 0.05, ** *p* < 0.01, *** *p* < 0.001.

Secretory phosphoprotein 1 (SPP1), also called osteopontin (OPN), is a multi-functional secreted glycoprotein that exists in the extracellular matrix. This molecule is synthesized and secreted by various tissues and cells, regulating mineralization, bone remodeling, inflammation, and immune responses. SPP1 can also bind with matrix molecules such as fibronectin and collagen to affect extracellular matrix remodeling, playing an important role in bone development, growth, and fibrosis [3]. The SPP1 signaling pathway was activated and increased between the macrophages, B cells, hepatocytes, hepatic stellate cells, macrophages, and T-cells in clusters 1 and 2 at 6 w after infection. The expression of the SPP1 gene was elevated in clusters 1 and 2 of the macrophages, and its receptor, CD44 (Supplementary Figure S5A), was also upregulated in all the types of cells (Figure 6A–C). The qRT-PCR results revealed that the SPP1 expression and CD44 increased in the liver at 6 w after infection (Figure 6D). 

The IGF-1 signaling pathway is upregulated between hepatocytes, macrophages, and neutrophils. The gene encoding IGF-1 was more highly expressed in the cluster 2 macrophages at 6 w after infection, and its receptor, IGF-1r (Supplementary Figure S5B), was more highly expressed in the neutrophils and macrophages (Figure 6A–C). The IGF-1 signaling pathway was significantly weakened at 10 w after infection. The qRT-PCR results confirmed that the expression of IGF-1 and IGF-1r increased at 6 w after infection (Figure 6D). IGF-1 has a structure similar to that of insulin and is a low-molecular-weight peptide that mediates growth hormones, regulating cell differentiation, proliferation, and apoptosis [16]. As a metabolic factor, this molecule plays a key role in regulating blood glucose and lipid levels, improving insulin beta-cell function and the development of type 2 diabetes [17].

Visfatin, also known as nicotinamide phosphoribosyltransferase (Nampt), was increased between the macrophages and hepatocytes 6 w after schistosome infection. Visfatin was more highly expressed in the macrophages at 6 w after infection, and its receptor, insulin receptor (Insr) (Supplementary Figure S5C), was more highly expressed in the hepatocytes and macrophages (Figure 6A–C). The qRT-PCR results confirmed that the expression of visfatin and Insr increased in the livers of the mice at 6 w after infection (Figure 6D). Visfatin can be secreted by macrophages, adipocytes, and skeletal muscle cells and has insulin-like functions. This molecule affects blood glucose levels and regulates glucose metabolism by increasing the glucose uptake capacity in adipose and muscle tissues and reducing liver glucose release. In addition, visfatin participates in processes such as lipid metabolism, oxidative stress, the inflammatory response, and endothelial dysfunction [18].

## 4. Discussion

Schistosomiasis is a zoonotic parasitic disease that poses a significant threat to human health. Schistosomes parasitize the inferior mesenteric vein, producing eggs that enter the liver with the blood flow, deposit in the liver, and induce an immune response in the host. Egg granulomas form in the liver, and the subsequent fibrosis poses a considerable threat to human health [19,20]. In the propagation of the disease, pathogens spread at different rates in different tissues, with heterogeneous properties. The heterogeneity in the spatial distribution of cells plays a key role in the spread of bacteria. The presence of spatial inhomogeneity, characterized by elevated local cell concentrations in clusters, has been observed to result in the initiation of events occurring at an earlier stage [21]. Single-cell sequencing technology provides convenience for the study of cell heterogeneity.

During the process of schistosome infection, the cells in liver tissue exhibit high heterogeneity. Research has shown [22] that many inflammatory cells, including eosinophils, macrophages, neutrophils, and fibroblasts, which are found in acute granulomas, appear around the eggs in the livers of mice at 6–8 w after infection. The area of the egg granulomas decreased in the livers of the mice at 10 w after infection, with the eggs disintegrating and rupturing and many macrophages infiltrating and fibroblasts appearing, indicating chronic granulomas. To observe the heterogeneity of cells during the acute and chronic phases and their effects on the egg granulomas and fibrosis, we used scRNA-seq to analyze the changes and interactions of immune cells in the livers of the mice before infection (0 w) and at 6 w and 10 w after infection in this study, which is helpful for identifying key cellular and molecular events that promote egg granulomas and fibrosis and discovering new drug targets against schistosomiasis.

In this experiment, MobiNova-100 for scRNA-seq was used, which has been applied in heterogeneity studies of various disease models, such as tumors [23,24,25], liver fibrosis caused by carbon tetrachloride or a high-fat diet [26], and ulcerative colitis [27]. This experimental method uses a molecular barcode for single-cell recognition, utilizing unique labeling and water-in-oil technology. By optimizing the design of the microfluidic chips and instrument control, a high cell capture rate is achieved [28,29,30,31].

In this study, 11 major categories of cells were identified through dimensionality reduction clustering and classic cell marker genes. The T-cells (Figure 2), macrophages (Supplementary Figure S6), and B cells (Supplementary Figure S7) were subsequently clustered and identified into different subtypes. The composition or proportion of cells before and after infection was significantly different. The immune cells were predominantly macrophages at 6 w post-infection, while neutrophils, macrophages and T-cells were predominant at 10 w post-infection. The proportions of effector CD4 T-, NK, naïve T-, and γδ T-cells decreased after infection, whereas the proportions of effector CD8 T-, Th1, Th2, and Treg cells increased. The Th1 cells proportion first increased and then declined, while the Th2 and Treg cells proportions gradually rose with the progression of infection. The results confirmed the heterogeneity of liver cells and T-cells in the liver during schistosome infection, which was helpful in providing more accurate diagnosis and treatment directions for liver fibrosis of schistosomiasis.

Through intercellular communication analysis, inflammatory signaling pathways, such as the CCL, TNF, CXCL, complex, and ALCAM pathways, were found to increase and enhance cellular communication after infection. Among them, CCL5-CCR5, CCL4-CCR5, CCL6-CCR2, and CXCL2-CXCL2R increased after infection, with the CCL5-CCR5 signaling pathway between macrophages and T-cells predominating at 6 w post-infection and the CCL6-CCR2 signaling pathway between macrophages at 10 w. Research has shown that CCL5-CCR5 participates in inflammatory responses through the downstream NF-κB pathway [32]. Additionally, CCL5-CCR5 has been demonstrated to facilitate the adhesion and migration of diverse T-cell subsets in the context of immune responses [33]. CCL5 has been shown to be necessary for T infiltration and activation, and activated T-cells release more inflammatory factors, exacerbating inflammatory damage [34]. In this study, CCL5 was expressed in all the T-cell subpopulations. Therefore, the infiltration and activation of T-cells in egg granulomas after 6 w of schistosomiasis infection may depend on the CCL5-CCR5 signaling pathway between macrophages and T-cells, which promotes the inflammatory response of egg granulomas. In addition, 10 w after schistosome infection, chemokines in the liver are regulated, mainly by the CCL6-CCR2 signaling pathway in macrophages. It has been confirmed that CCL6 contributes to the promotion of M2 macrophage polarization and the acceleration of wound healing through the regulation of the PI3 kinase/Akt signaling pathway [35]. At 10 w after schistosome infection, the PI3K/Akt signaling pathway was enriched in the two groups of M2 macrophages, indicating that CCL6 promoted macrophage polarization toward the M2 phenotype and played a crucial role in damage repair at the later stage of infection. Previous studies have confirmed that CXCL2 is a chemotactic factor for neutrophils. CXCL2^+^ macrophages recruit neutrophils to the aging liver via the CXCL2-CXCR2 signaling pathway and stimulate the formation of neutrophil extracellular traps by secreting IL-1β and TNF-α, leading to age-related liver injury [36]. The CXCL2-CXCL2R signaling pathway between macrophages and neutrophils was significantly activated and elevated in the livers of mice at 6 and 10 w post schistosome infection, indicating that the CXCL2 secreted by macrophages in the livers of mice after schistosome infection attracted and activated neutrophils through the CXCL2-CXCL2R signaling axis, playing an important role in the inflammatory damage and repair of egg granulomas.

Eggs deposited in the liver induce granulomatous inflammation and fibrosis mediated by CD4^+^ Th cells in the host. Our study revealed that the signaling between the costimulatory signaling molecules CD80 on neutrophils and CD28 on the surface of T-cells was increased in the livers of mice after schistosome infection, as determined by scRNA-seq, and that T-cells were activated. However, CD274, also known as programmed death ligand 1 (PD-L1), is also expressed on the surface of T-cells and may have a negative regulatory role. The sequencing results revealed that, compared with those in healthy mice, the proportion of T-cells, especially CD4^+^ T-cells, in the liver decreased at 6 and 10 w after schistosome infection. Currently, the role of T-cells in egg granulomas and fibrosis caused by schistosome infection is mainly focused on CD4^+^ Th cells, and little is known about the role of CD8 T-cells. The present study of the single-cell transcriptome revealed that the proportion of CD8^+^ T-cells was also elevated, suggesting that they may also be involved in liver injury and fibrosis caused by schistosome infection. The literature reported as early as in 1997 [37] that schistosome infection by *Schistosoma mansoni* resulted in the generation of a type 1 CD8^+^ cell response, whose main function was to regulate the Th2 response, thereby regulating the formation of egg granulomas and fibrosis. The role of CD8^+^ T-cells in egg granulomas and fibrosis needs to be further studied in depth.

Our study also revealed that schistosome infection leads to activation of and an increase in collagen signaling pathways, as well as the SPP1 signaling pathway, in liver fibrosis via scRNA-seq. Previous research has reported that the levels of SPP1 in the serum of mice and humans infected with schistosomes are elevated [38]. Furthermore, soluble egg antigen (SEA) stimulates macrophages to secrete SPP1, which stimulates hepatic stellate cell activation and collagen deposition and promotes the development of liver fibrosis. SPP1 is an important drug target for controlling hepatic pathological damage in schistosomiasis [39]. In addition, SPP1 is a marker for calcium macrophages, acinar-shaped mononuclear macrophages distinguished from rounded “omelette”-shaped GM-CSF macrophages and elongated M-CSF macrophages, and is characterized by the overproduction of SPP1 and a strong proinflammatory cytokine response [40]. SPP1 not only plays an important role in inflammation and fibrosis but also participates in bone strength and bone remodeling processes. High expression of SPP1 is a risk factor for osteoporosis, as it positively regulates osteoclasts and inhibits bone mineral deposition [41]. Schistosome infection leads to abnormal bone loss mediated by osteoclasts, and further studies have confirmed that Tfh cell subsets may cause bone loss by releasing soluble RANKL [42]. The increase in SPP1 observed in our study after schistosome infection may be another important cause of bone loss, which needs to be further confirmed.

In addition to its pathogenic effects, recent studies have confirmed that schistosome infection can be used to treat autoimmune and inflammatory diseases [43]. Studies have reported significant reductions in insulin resistance and blood glucose in type 2 diabetic Leprdb/db mice given SEA of *S. japonicum* twice weekly for 6 w [44]. The prevalence rate of diabetes among people with previous schistosome infection was lower [45]. Schistosome infection can alleviate or prevent the development of type 1 diabetes (T1D) [46]. The above studies confirmed that schistosome infection can be used to treat type 1 and type 2 diabetes, and the specific mechanism may be related to the expansion of Th2 and Treg cells caused by schistosome infection [47,48]. Our study demonstrated an elevation in the levels of the IGF-1 and visfatin signaling molecules secreted by macrophages in the livers of mice after schistosome infection, both of which are involved in regulating blood glucose metabolism. Decreases in the serum level of IGF1, similar to insulin, are positively related to the occurrence of type I and 11 diabetes [49,50,51]. The overexpression of IGF1 in vivo significantly reduces the incidence of diabetes, with normal islet beta-cell function and normal insulinemia, indicating that IGF1 regulates islet autoimmunity in NOD mice [52]. Visfatin exerts insulin-like effects by binding to Insr-1 and has hypoglycemic effects [53]; low levels of visfatin have been detected in the serum of individuals with type 2 diabetes [54,55]. Therefore, the increased IGF-1 and visfatin after schistosome infection may be important for the treatment of diabetes. In addition, visfatin is closely related to cardiovascular diseases. Visfatin plays a protective role in atherosclerosis, myocardial infarction and hypertension by reducing endothelial dysfunction, promoting cell proliferation and migration, mediating the secretion of anti-inflammatory factors, and inhibiting the mitochondrial permeability transition pore [56,57]. Schistosome infection may play a protective role in cardiovascular diseases and deserves further study.

It should be noted that the small sample size is the limitation of this study. However, histopathological sections, qRT-PCR and flow cytometry analysis were employed to validate the scRNA-seq results with multiple mouse samples. Our study provided the comprehensive single-cell transcriptome landscape of liver in mice during the progression of schistosome infection. However, the role of elevated CCL5-CCR5, CCL6-CCR2, SPP1, IGF-1 and visfatin signaling pathways needs to be studied later. Considering the further functional research, C57BL/6 mice were selected for the scRNA-seq analysis, which are most commonly used as the maternal parent of gene knockout mice. The other mice strains, such as BALB/C mice, would be used to validate the results in the future.

## 5. Conclusions

In conclusion, our study demonstrated remarkable variations in the composition or proportion of immune cells in the livers of mice during the progression of *S. japonicum* infection via scRNA-seq. Moreover, the elevated CCL5-CCR5 signaling pathways in acute phase and CCL6-CCR2 signaling pathways in the chronic phase may be the drug target for liver injury and fibrosis caused by schistosome infection, respectively. In addition to the various subsets of CD4^+^ T-cells, CD8^+^ T-cells are also involved in the liver injury and fibrosis of schistosomiasis. Elevated SPP1, IGF-1 and visfatin in the liver after schistosome infection may play a role in bone metabolism and insulin metabolism, which needs to be further studied. Overall, we provide the comprehensive single-cell transcriptome landscape of mouse livers in response to schistosome infection and delineate the key cellular and molecular processes involved in schistosome infection-induced liver injury and fibrosis, which is helpful for identifying new drug targets for treating schistosomiasis and providing evidence of the role of schistosome infection in metabolic diseases.

## Data Availability

The original contributions presented in the study are included in the article/Supplementary Material; further inquiries can be directed to the corresponding author.

## Supplementary Materials

The following supporting information can be downloaded at https://www.mdpi.com/article/10.3390/cimb46110707/s1, Figure S1: Heatmap of the marker gene expression in the cells of different clusters; Figure S2: Heatmap of the marker gene expression in the different T-cell subtypes; Figure S3: A Distribution of the CCL ligands and receptors in the liver at 6 and 10 w after schistosome infection. B Violin plot of the expression distribution of the CCL signaling pathway-related genes; Figure S4: A Distribution of the CXCL ligands and receptors in the livers of schistosome-infected mice at 6 and 10 w. B Violin plot of the expression distribution of the CXCL signaling pathway-related genes; Figure S5: A Distribution of the SPP1 ligands and receptors in the liver at 6 w after schistosome infection; B Distribution of the IGF1 ligands and receptors in the liver at 6 w after schistosome infection; C Distribution of the visfatin (Nampt) ligands and receptors in the liver at 6 w after schistosome infection; Figure S6: Dimensionality reduction of 5374 macrophages shown by clustering UMAP plot (stained by cell type and infection time); Figure S7: Dimensionality reduction of B cells shown by clustering UMAP plot (stained by cell type and infection time); Table S1: qPCR primer sequences.
