# Supplementary material for: Single-Cell Analysis Reveals the Cellular and Molecular Changes of Liver Injury and Fibrosis in Mice During the Progression of Schistosoma japonicum Infection
Source: Curr Issues Mol Biol. 2024 Oct 23;46(11):11906–26. doi: 10.3390/cimb46110707 (PMC11592686; doi:10.3390/cimb46110707)
Supplement: Supplementary file 1 [file cimb-46-00707-s001.zip › cimb-3245978-supplementary.pdf]

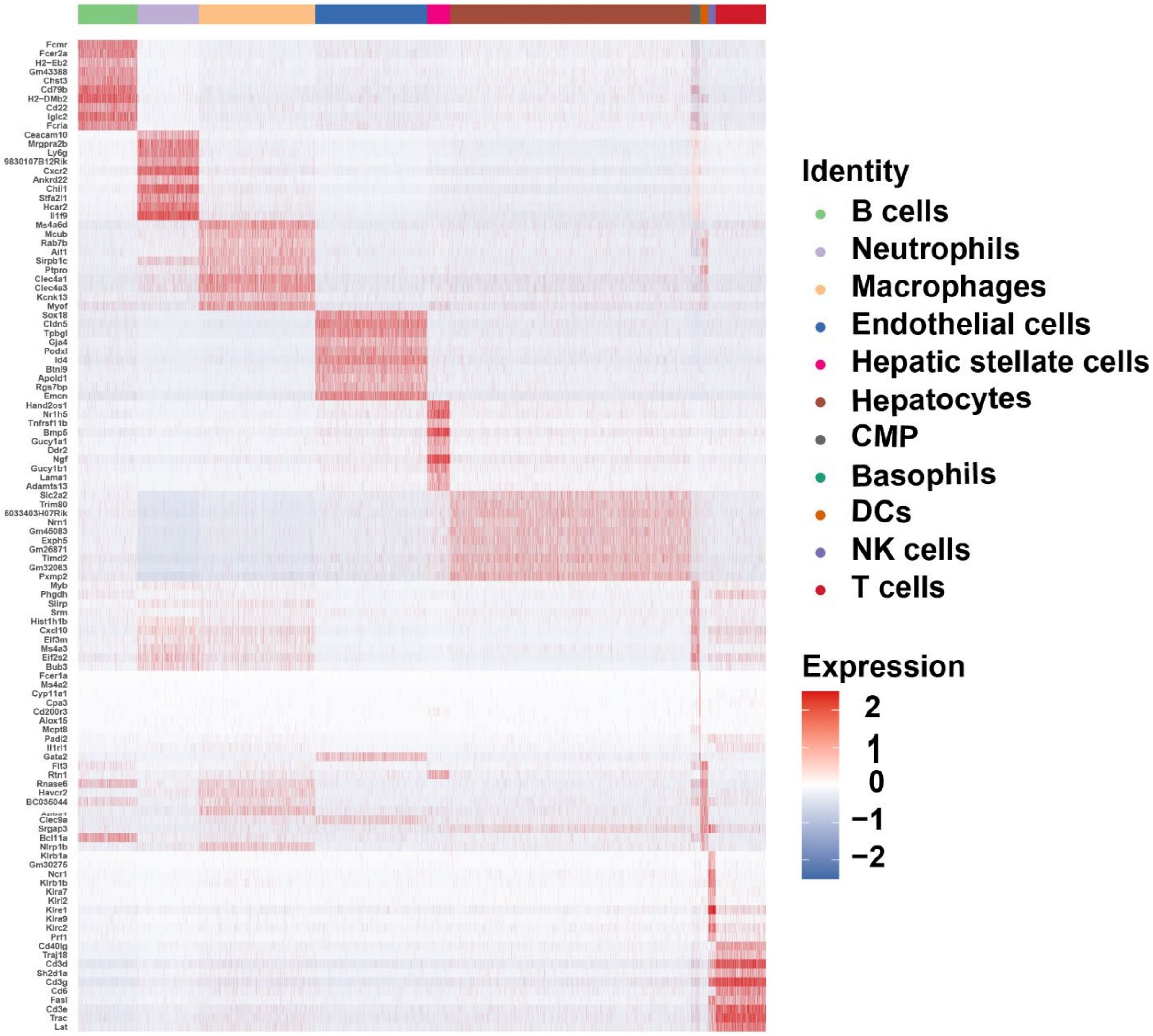

Supplementary Figure S1. Heatmap of marker gene expression in cells of different clusters.

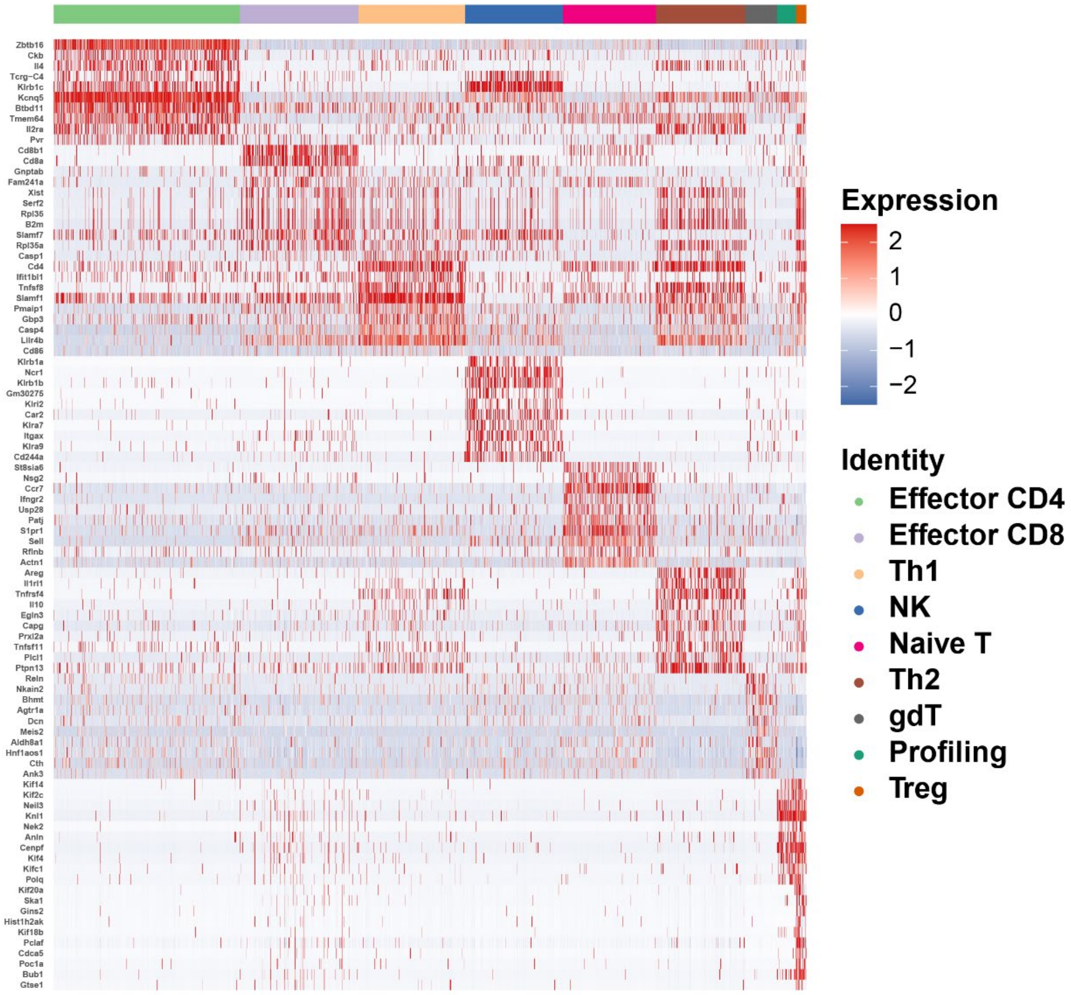

Supplementary Figure S2. Heatmap of marker gene expression in different T-cell subtypes.

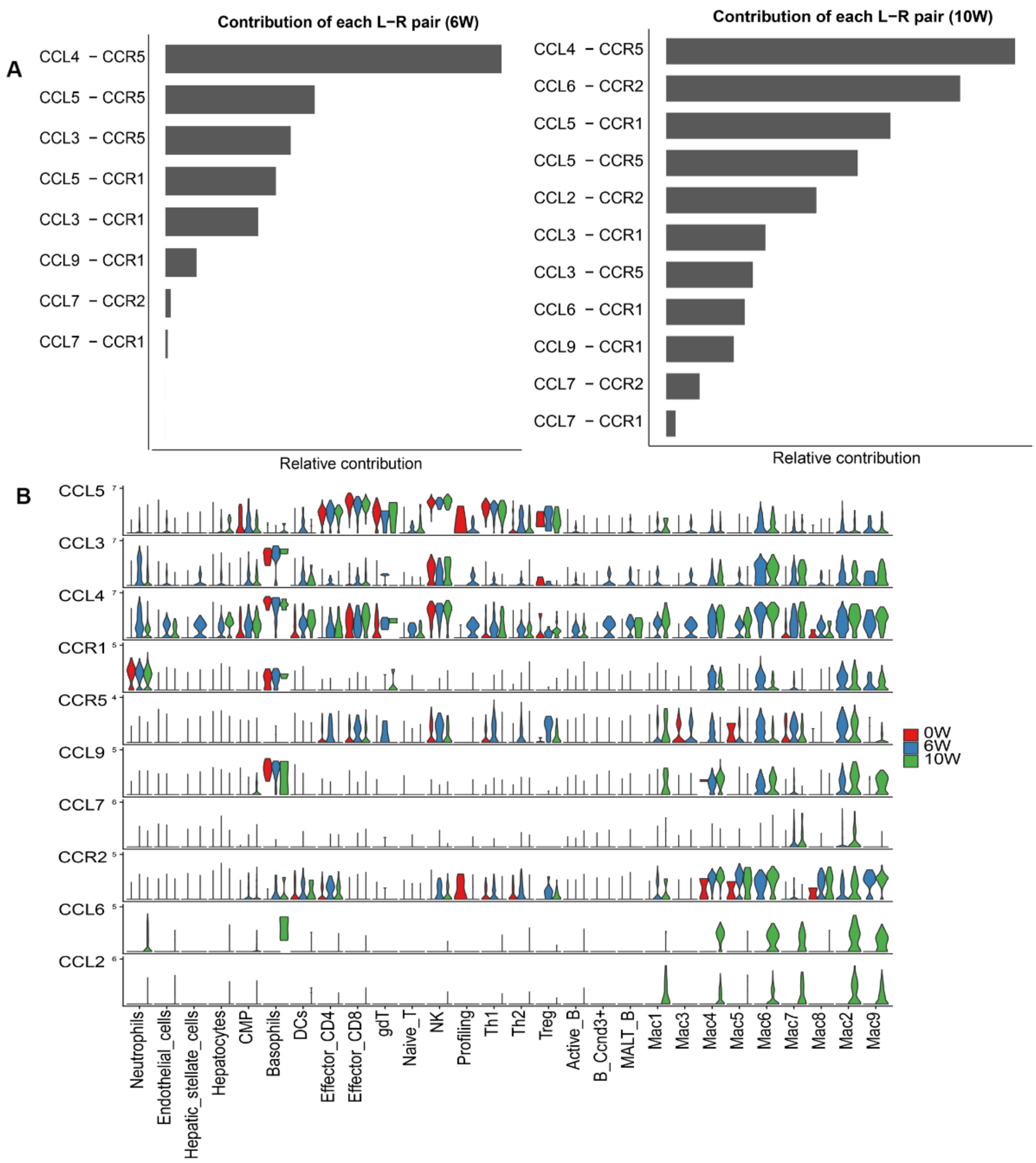

Supplementary Figure S3. A Distribution of CCL ligands and receptors in the liver at 6 and 10 w after schistosome infection. B Violin plot of the expression distribution of CCL signaling pathway-related genes.

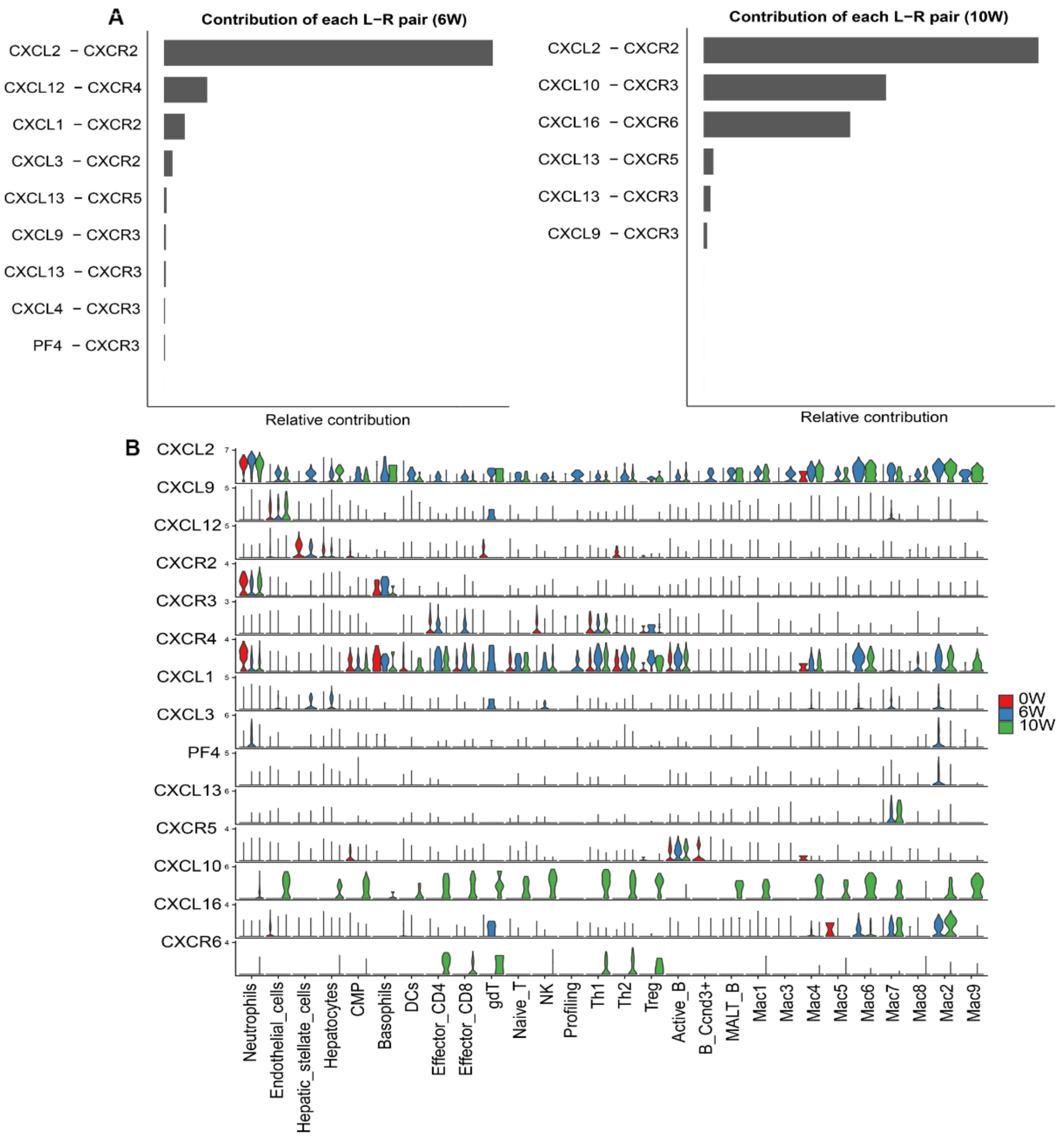

Supplementary Figure S4. A Distribution of CXCL ligands and receptors in the livers of schistosome-infected mice at 6 and 10 w. B Violin plot of the expression distribution of CXCL signalling pathway-related genes.

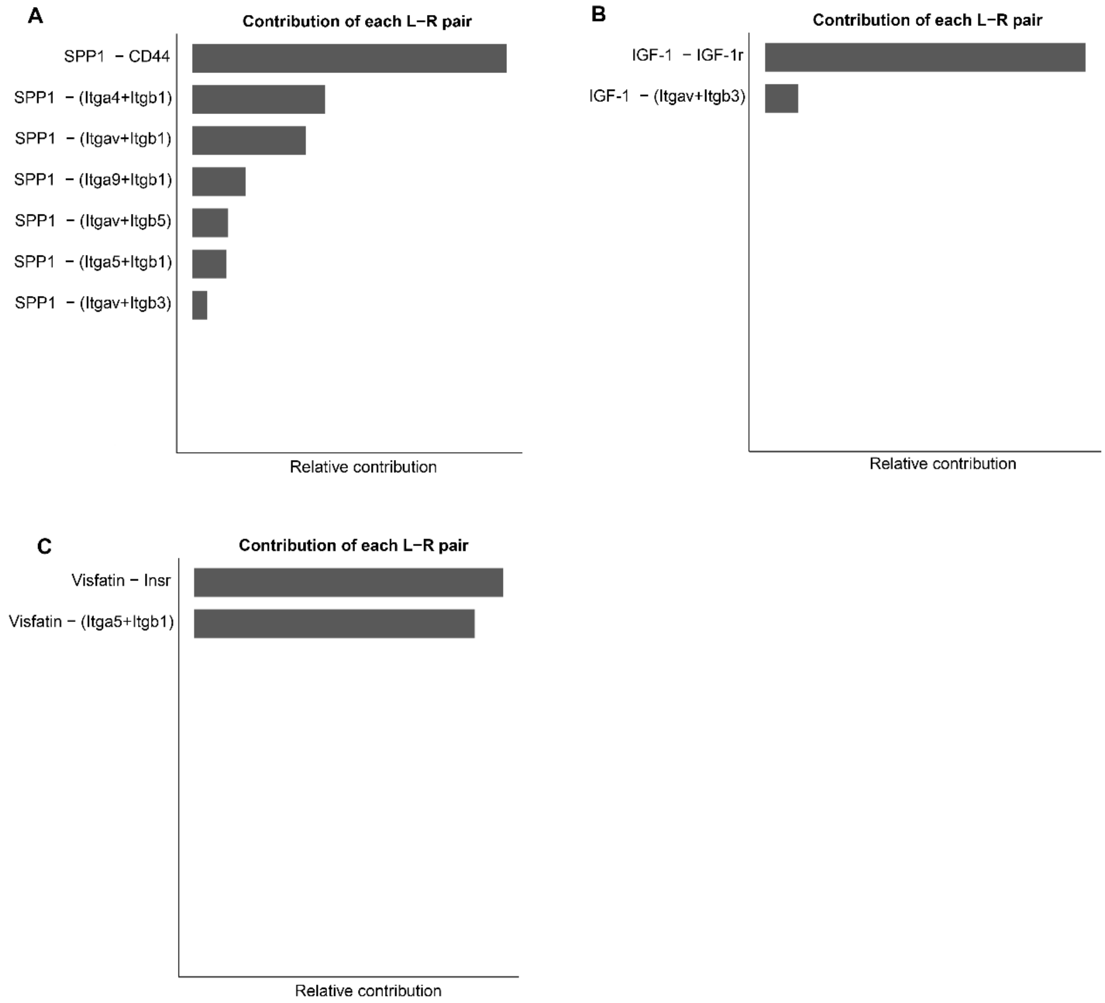

Supplementary Figure S5. A Distribution of SPP1 ligands and receptors in the liver at 6 w after schistosome infection; B Distribution of IGF1 ligands and receptors in the liver at 6 w after schistosome infection; C Distribution of Visfatin (Nampt) ligands and receptors in the liver at 6 w after schistosome infection.

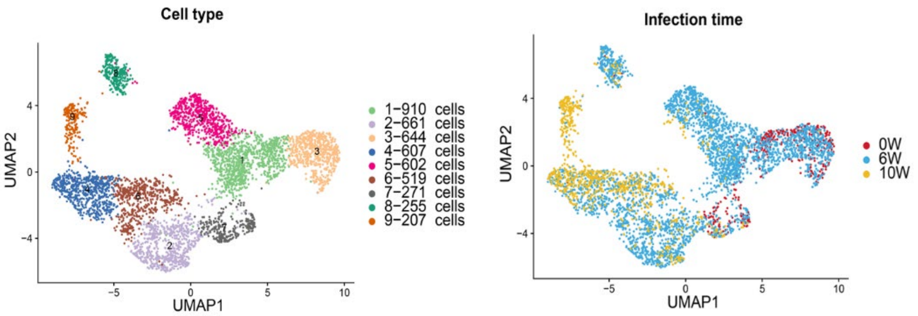

Supplementary Figure S6. 5374 macrophages dimensionality reduction clustering UMAP plot (stained by cell type and infection time).

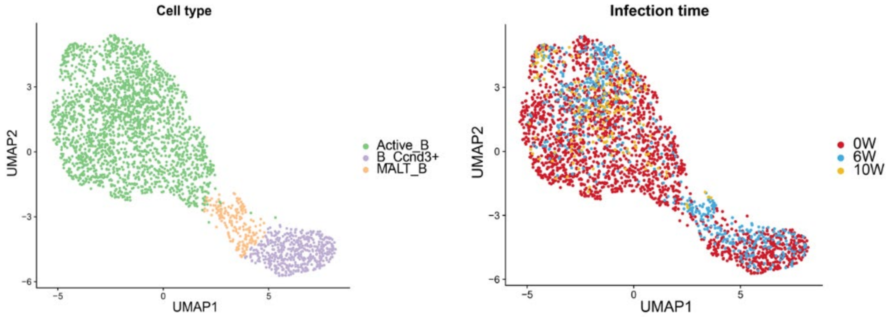

Supplementary Figure S7. 2723 B cells dimensionality reduction clustering UMAP plot (stained by cell type and infection time).

Supplementary Table S1 qPCR primer sequences

	**Sequences (5'-3')**
CCL4	Forward: TTCCTGCTGTTTCTCTTACACCT<br>Reverse: CTGTCTGCCTCTTTTGGTCAG
CCL5	Forward: GCTGCTTTGCCTACCTCTCC<br>Reverse: TCGAGTGACAAACACGACTGC
CCL6	Forward: TTATCCTTGTGGCTGTCCTTG<br>Reverse: TGGAGGGTTATAGCGACGAT
CCR5	Forward: TCGAGTGACAAACACGACTGC<br>Reverse: TTTTCAAGGGTCAGTTCCGAC
CCR2	Forward: GGAGAAAAGCCAACTCCTTC<br>Reverse: TCTCTGAGTGGCATGGGACA
CXCL2	Forward: GGAAGCCTGGATCGTACCTG<br>Reverse: TGAAAGCCATCCGACTGCAT
CXCR2	Forward: CTACTGCAGGATTAAGTTTACCTC<br>Reverse: ATGGGCAGGGCCAGAATTAC
CD80	Forward: GCCTCGCTTCTCTTGGTTG<br>Reverse: TTACTGCGCCGAATCCTG
CD28	Forward: TCCTACAACCTTCTCGCA

	Reverse: CGGGGAGTCATGTTCATG
CD274	Forward: GCTCCAAAGGACTTGTACGTG
	Reverse: TGATCTGAAGGGCAGCATTTC
SPP1	Forward: AGAATGCTGTGTCCTCTGAAG
	Reverse: TCGTCATCATCGTCGTCCA
CD44	Forward: GAATTCTGCGCCCTCGGTT
	Reverse: CTGCCTCAGTCCGGGAGATA
IGF-1	Forward: GGACCGAGGGGC-TTTTACTT
	Reverse: TCCGGAAGCAACACTCATCC
IGF-1r	Forward: AAACGCTGACCTCTGTTACCTCTC
	Reverse: GCGGATGAAGCCTGATGGAC
Visfatin	Forward: CTCCACTGCCTTGCTCTTATTT
	Reverse: CCGCTGGTGTCCTATGTAAA
Insr	Forward: ATGGCAACATCACACACTACC
	Reverse: AGCCCTTTGAGACAATAATCC
GAPDH	Forward: AACTTTGGCATTGTGGAAGG
	Reverse: CCCTGTTGCTGTAGCCGTAT
